# Influencing Factors of Understanding COVID-19 Risks and Coping Behaviors among the Elderly Population

**DOI:** 10.3390/ijerph17165889

**Published:** 2020-08-13

**Authors:** Zhonggen Sun, Bingqing Yang, Ruilian Zhang, Xin Cheng

**Affiliations:** 1 School of Public Administration, Hohai University, Nanjing 211100, China; sunzhonggen@hhu.edu.cn (Z.S.); yangbingqing@hhu.edu.cn (B.Y.); 1707020105@hhu.edu.cn (X.C.); 2 Sustainable Minerals Institute, University of Queensland, Brisbane 4072, Australia

**Keywords:** COVID-19, elderly population, risk cognition, behavior, influencing factors

## Abstract

It is known that the elderly population has weak immune functioning and is a susceptible and high-risk group with respect to the current coronavirus disease 2019 (COVID-19) epidemic. In this study, to understand the influencing factors of COVID-19-related risks and coping behaviors of elderly individuals with respect to COVID-19 and to provide a basis for taking corresponding protective measures, a questionnaire survey was applied to an elderly population. One-way analysis of variance (ANOVA) and linear regression analysis were used to explore the influencing factors of the level of understanding of COVID-19 risks among the elderly population. Additionally, the chi-square test and logistic regression analysis were used to explore the influencing factors of the elderly population’s protective behaviors against COVID-19. This study found: (1) The sex, age, and self-care ability of elderly individuals were significantly correlated with their level of understanding of COVID-19, and that those who were female, were of a younger age, or had better self-care ability had higher levels of understanding; (2) The sex, place of residence, and level of understanding of COVID-19 among the elderly individuals were significantly correlated with their protective behaviors, e.g., those who were women, had high levels of understanding, and lived in cities were more likely to have good behaviors; (3) Elderly individuals’ assessments of COVID-19 information provided by the government were significantly correlated with their protective behaviors—those who had a positive evaluation of relevant information provided by the government were more likely to develop protective behavior. The conclusions of this study show that it is crucial to implement COVID-19 prevention and control measures in the elderly population. Society, communities, and families need to increase their concerns about the health and risk awareness of the elderly individuals.

## 1. Introduction

### 1.1. Background

In December 2019, the first case of coronavirus disease 2019 (COVID-19) infection was found in Wuhan, Hubei Province, China. Subsequently, the COVID-19 outbreak, which has community transmission characteristics [1], broke out in Wuhan and spread rapidly across China, causing great concern domestically and internationally. As of June 14, 2020, a total of 84,729 cases of COVID-19 have been reported nationwide, with 4645 deaths; additionally, 7,690,708 confirmed cases have been reported worldwide [2], with the number of infections rising rapidly worldwide. The epidemic situation in China is approaching its end, but the pandemic situation around the world has just begun, and currently, it is severe. The attention of the academic community has focused on urging all of society to actively take measures to prevent the disease.

With declining cognitive ability [3], poor physiological function and physical fitness, and low immune function [4], elderly individuals with underlying diseases have significantly higher susceptibility than other populations [5,6]. In addition, due to the decline in cognitive ability, the elderly population is prone to anxiety [7], resulting in psychological instability. In the context of the lack of effective symptomatic drugs, the elderly population may suffer from more significant health risks in the face of sudden illnesses [8]. Most countries worldwide have an aging society. It is estimated that the global proportion of elderly individuals over 65 years old will increase from 11% in 2019 to 16% in 2050. Preventing COVID-19 infection in the elderly population and reducing new cases and deaths will play a key role in overcoming the current epidemic [9]. It is important to enable the elderly population to objectively understand COVID-19 risks and take scientific prevention and control measures. Therefore, research on the prevention of COVID-19 in the elderly population is urgently necessary.

Risk cognition is an individual’s subjective feelings, experience, and understanding of various objective risks that exist in the external environment [10]. In this paper, the understanding level of COVID-19 refers to the understanding of COVID-19-related knowledge of risks [11,12]. We measured the understanding level of COVID-19 risks from three dimensions: empirical features, ethical characteristics, and prevention and control measures. Previous studies have shown that improving the public’s understanding of COVID-19 risks and promoting its positive protective behavior can effectively prevent the epidemic situation and reduce the risk of infection with COVID-19 [13,14]. At present, the academic community has conducted a series of studies on the influencing factors of understanding of COVID-19-related risks and behavior of the elderly population with respect to sudden illness. Cao proposed that age, household registration location, educational level, and occupational distribution were the main factors affecting the coping behavior of elderly patients [15]; Wei proposed that a sound family support system affected elderly individuals’ awareness of sudden illness and was an essential factor of coping behaviors [16]; Xu proposed that gender and physical health were the main influencing factors of the elderly population’s understanding of disease [17]. Due to the mixed results yielded by different studies, determining the factors that influence the understanding of disease and behavior of the elderly population in this new context is vital and warrants further study to develop targeted intervention programs and policies, aiming to help reduce the risk of infection in this population.

In the current study, we investigate the cognitive level and behavioral status of elderly among individuals over the age of 60 in China, analyze their cognitive and behavioral influencing factors, and proposed corresponding prevention and control recommendations for the elderly population.

### 1.2. Hypotheses

Risk cognition theory is the basis of this article. As proposed by Paul Slovic [18], risk cognition refers to an individual’s feelings and understanding of various objective risks existing in the outside world, and it emphasizes the impact of the individual’s experience on intuitive perception and subjective experience. Subsequently, risk cognition has been used by other researchers to study natural, economic, and technological risks. An individual’s risk perception will be affected by the individual’s own characteristics, social, cultural and institutional factors. If an individual holds a strong initial viewpoint, it is generally difficult to change, which directly affects the individual’s understanding and acceptance of subsequent information [19]. Subjective perceptions better explain the irrational behavior caused by individuals’ cognitive deviations, and they are effective predictors of health problem decision-making and health behavior interventions [20].

In terms of the overall public awareness of COVID-19, the public has paid close attention to the spread of the disease and has a high awareness of it. In particular, women, those belonging to older age groups (middle-aged), people living in cities, people with medical backgrounds [21], and people with higher educational levels have higher awareness rates [11]. From the perspective of the public’s understanding of influenza, some scholars have found that there are differences in the awareness rates of influenza between the urban population and the rural population, with higher cognition levels among urban residents [22]. From the perspective of the cognitive ability of the elderly population, self-care ability and participation in leisure activities significantly affect cognitive functioning, and elderly individuals with high self-care ability also have better understanding levels [23]. From the perspective of the elderly population’s knowledge of pneumonia and other diseases, some scholars have investigated knowledge regarding pneumonia (pneumococcus) in the elderly population, finding that pneumonia awareness was different among survey subjects with different educational levels and levels of monthly disposable incomes. Older people with higher education levels and higher levels of monthly disposable income had a higher awareness rate [24]. Taking Nanjing city as the survey scope to analyze elderly individuals’ knowledge of pneumonia, one study found that the level of cognition among such individuals was higher in females and in those with a history of respiratory diseases [17]. Based on the above analyses, the following hypotheses (H1–H5) are proposed:

**Hypothesis** **1.** *Sex is associated with the level of understanding of COVID-19, and female elderly individuals have a higher rate of understanding.*

**Hypothesis** **2.** *Age is negatively correlated with the level of understanding of COVID-19.*

**Hypothesis** **3.** *Educational level is positively associated with the level of understanding of COVID-19.*

**Hypothesis** **4.** *Self-care ability is positively correlated with the level of understanding of COVID-19.*

**Hypothesis** **5.** *Place of residence is associated with the level of understanding of COVID-19, and urban residents have a higher rate of understanding.*

Some scholars have surveyed and investigated the level of personal protection against influenza among residents in Guangzhou, finding that there were differences in the precautionary measures taken by people of different sex. The proportion of female respondents who took measures to prevent influenza was higher than that of male respondents, and women paid more attention to personal protection [23]. Some studies have suggested that younger people are more efficient in receiving correct information and translating that information into action. The higher the public’s awareness is of infectious diseases, the higher the correctness and timeliness of adopting healthy behaviors [11]. Some scholars analyzed the influencing factors of public health-related behaviors during the severe acute respiratory syndrome (SARS) epidemic and found that during the SARS outbreak in China in 2003, establishing of public health behaviors was influenced by urban–rural differences and educational levels. The health behaviors of rural residents and people with a low educational level were poor [25]. Taking the rural elderly population as research subjects, one study found that rural elderly populations were more likely to exhibit health hazards, such as group aggregation [26]. Some scholars analyze and simulate the mentality of people during a crisis period (taking SARS as an example), finding that the higher the public’s understanding of prevention and treatment, the better its behavior indicators would be in the event of a major emergency [27]. Based on the above analyses, the following hypotheses are proposed:

**Hypothesis** **6.** *Sex is significantly associated with protective behavior, and female elderly individuals are more likely to take good protective actions.*

**Hypothesis** **7.** *Age is negatively correlated with protective behaviors, and younger people are more likely to have good behaviors.*

**Hypothesis** **8.** *Place of residence is significantly associated with protective behaviors, and urban residents are more likely to have good behaviors.*

**Hypothesis** **9.** *The level of understanding of COVID-19 is positively correlated with protective behaviors. Those with higher levels of understanding are more likely to have good behaviors.*

To respond to emergency events, related research on public behavior decision-making and its influencing factors has found that by providing open, transparent, and effective information, the government can increase public trust in the government, reduce group behaviors, promote rational public behavior, and reduce the uncertainty of the emergency response [28]. Consequently, the public’s evaluation of the information provided by the government regarding COVID-19 can reflect whether the government has provided an effective supply of information that the public deems satisfactory, thus affecting the public’s behavior in the face of the epidemic. Based on the above analysis, the following hypothesis is proposed:

**Hypothesis** **10.** *Participants’ evaluations of government-provided COVID-19-related information are significantly correlated with their protective behaviors, and positive evaluators are more likely to exhibit good behaviors.*

## 2. Materials and Methods

### 2.1. Data Collection

The target of the sampling survey was elderly individuals over 60 years old in China, and the inclusion criterion of study objects was belonging to the population aged 60 or older [4]. This investigation used snowball sampling to invite the elderly participants to complete a questionnaire online. Ten elderly subjects were selected based on factors such as sex, age, educational level, and place of residence. After the respondents completed the questionnaire online, they were asked to provide other subjects belonging to the target population under study. We used this method to obtain a large number of samples. To control the questionnaire quality, the same IP address could answer only once. Private information, such as individuals’ names, was not involved in the questionnaire, and sensitive language was avoided. The questionnaire contained a total of 40 questions. Based on the fastest speed of completing one question every three seconds, the responses were considered invalid if the time to complete the questionnaire was less than 120 s.

The questionnaire was available for 5 days, and a total of 545 were collected. After screening, there were 508 valid questionnaires, for an effective rate of 93.21%. The proportion of respondents from Hubei Province was 65.7%, and in the same period, the number of people infected with COVID-19 in Hubei Province accounted for 81.5% of the total number of people infected China. The geographical distribution of the investigated participants was close to that of the COVID-19-infected population, and the samples were well representative.

### 2.2. Variables

#### 2.2.1. Level of Understanding of COVID-19 Risks among the Elderly Individuals

Taking the understanding level of COVID-19 among the elderly individuals as the dependent variable, sex, age, educational level, self-care ability, and place of residence were used as independent variables.

Demographic characteristics were described by categorical variables, and in this paper, the level of understanding risk refers to the understanding of COVID-19-related risk knowledge and was measured by the number of correct COVID-19-related answers by the participants. There were 7 questions related to the level of understanding of COVID-19; among them, there were 2 single-choice questions and 5 multiple-choice questions. Additionally, 1 point was given for a correct answer and 0 points for an incorrect answer; then, the understanding performance of each participant was calculated.

#### 2.2.2. Coping Behaviors of Elderly Individuals

Taking the protective behaviors adopted by the elderly individuals as the dependent variable, the participants’ sex, age, educational level, place of residence, awareness of COVID-19, and evaluation of relevant information provided by the government were the independent variables.

Demographic characteristics were described by categorical variables, and protective behaviors were assigned and scored as either good behaviors or poor behaviors. With a maximum score of 7, the levels of understanding were divided based on the average score: high and low levels of understanding were scores above and below the average, respectively. The evaluation of relevant information provided by the government was measured using a Likert scale and was evaluated along 3 dimensions: the timeliness, adequacy, and authenticity of information disclosure. Each question was assigned a value of 1 to 5 points. The higher the score was, the higher the evaluation (scores < 3.5 were considered negative evaluations, while scores ≥ 3.5 were considered positive evaluations).

There were eight questions regarding behavior proposed by Zhan et al. [29], and points were given based on the frequency of active responses (wearing a mask, washing hands, reducing interpersonal contact, etc.): never, 1 point; occasionally, 2 points; sometimes, 3 points; often, 4 points; always, 5 points The total score for each person was calculated, and 31 points, the average score for all the respondents, was used as a cutoff; scores < 31 points represented poor behaviors, while scores ≥ 31 points represented good behaviors (similar classification criteria were adopted in previous studies [30]).

### 2.3. Statistical Analyses

Statistical analysis was performed using SPSS (version 25.0; SPSS Inc., Chicago, IL, USA). Descriptive statistics were first used to analyze the essential characteristics of the elderly subjects and variables. Then, reliability and validity tests were used to analyze the consistency and validity of the measurement results.

Taking the COVID-19 understanding level as the dependent variable, and sex, age, educational level, self-care ability, and place of residence as independent variables, univariate analysis of variance (ANOVA) and multivariate linear regression analysis were performed. In multivariate linear regression analysis, sex and place of residence were virtual variables; understanding level was a continuous variable; and age group, education level, and self-care ability were ordinal variables, and were regarded as continuous variables. They were directly included in the regression equation.

Using coping behavior as the dependent variable, elderly individuals’ demographic characteristic, understanding level, and evaluation of relevant information provided by the government were classified into categorical variables and used as independent variables. The chi-square test was first used for univariate analysis, then variables with statistical characteristics (*p* < 0.05) in the chi-square test were selected for unconditional logistic multivariate regression analysis (α enter = 0.05, α delete = 0.10).

The logistic regression model was established as follows:

(1) $$logit\left(p\right)=ln\left(\frac{p}{1-p}\right)=\beta_{0}+\beta_{1}x_{1}+\beta_{2}x_{2}+\cdots +\beta_{k}x_{k}+\mu$$

where p represents the probability of success, $x_{i}\left(i=1,2,\cdots ,k\right)$ is the independent variable of the model, $\beta_{i}\left(i=0,1,\cdots k\right)$ is the parameter to be estimated, and *μ* is the random interference term. This article analyzed the impact of different demographic characteristics, such as elderly individuals’ sex, place of residence, COVID-19 understanding level, and evaluation of relevant information provided by the government, on the preventive behavior based on the abovementioned logistic regression model.

### 2.4. Ethical Approval

For this study, the consent of the University Behavioral and Social Sciences Ethical Review Committee to which the researcher belongs was obtained (Approval number: SPA number 20200211). All respondents were given information about the aim of the study and that the data would be treated as strictly confidential and that all answers would be anonymous.

## 3. Results

### 3.1. Variable Descriptive Statistics

#### 3.1.1. Sample Population Attributes

After excluding invalid questionnaires, 508 respondents were included in the study. Table 1 presents the basic demographic characteristics of the respondents. Among the objects of this survey, 221 were male, and 287 were female, accounting for 43.5% and 56.5%, respectively, of the total participants. Regarding age, the 60–70 age group accounted for the highest percentage, 47.0%. In terms of educational level, 186 people had attended primary school, accounting for 36.6%, and 123 had attended middle school, accounting for 24.2%; 71 people had attended high school, accounting for 14.0%, and 18 people had attended university, accounting for 3.5%. Regarding place of residence, 55.1% of the respondents lived in cities, and 44.9% lived in rural areas.

#### 3.1.2. Understanding Level of COVID-19

As seen in Table 2, “*n*” represents the number of people who answered this question correctly, and “%” represents the rate of correct answers. Among the related questions, the lowest rate of correct answers was for the main symptoms of COVID-19; only 15.6% of elderly participants answered this question correctly. The cognition rate of the etiology of COVID-19 was also low, at 28.1%. The average overall score was only 2.37, indicating a low overall level of understanding of COVID-19. Two participants (0.4%) answered all the questions correctly.

#### 3.1.3. Response Measures Taken by Elderly Individuals

Table 3 presents the measures taken by elderly individuals in response to COVID-19. The table shows that, in general, most elderly individuals had adopted positive epidemic prevention and control measures; however, a considerable number of elderly individuals had adopted negative measures (including acting as though the outbreak is not happening; i.e., no measures were taken, and their everyday life had not changed) to deal with it. Some elderly individuals used unproven preventive measures, including the intake of antiviral drugs.

#### 3.1.4. Elderly Individual’s Evaluation of COVID-19-Related Information Provided by the Government

(1) Regarding the primary source of COVID-19-related information, community/village cadre notifications (village broadcast notifications, home notifications) accounted for the most significant proportion of sources of epidemic information for elderly individuals, accounting for 63.98% of the total proportion, followed by verbal information from their children and television broadcasts. Evidently, local governments have performed well with respect to providing information pertaining to the epidemic situation to villages and households in general. (2) Based on the Likert scale, a mean between 1.0 and 2.4 indicated disagreement, a mean between 2.5 and 3.4 indicated neutrality, and a mean between 3.5 and 5.0 indicated agreement. Table 4 shows that elderly individuals’ average overall evaluation of COVID-19-related information provided by the government and media was 3.92, which was relatively high. The scores for the adequacy and authenticity of information disclosure were slightly higher than those for timeliness.

### 3.2. Reliability and Validity Tests

To verify the reliability and validity of the questionnaire, the information evaluation scale in the questionnaire was evaluated. The Cronbach’s alpha value was 0.867, i.e., higher than 0.7, indicating that the questionnaire had good consistency, and the Kaiser–Meyer–Olkin (KMO) value (0.738) and Bartlett’ s test of sphericity value (*p* = 0.000 (<0.001)) indicated good validity.

### 3.3. Analysis of the Influencing Factors of the Understanding of COVID-19 among the Elderly Individuals

#### 3.3.1. Univariate ANOVA of the Understanding of COVID-19 among the Elderly Individuals

The level of understanding of COVID-19 among the elderly individuals based on different characteristics is presented in Table 5. $\bar{x}\;\pm \;s$ refers to mean ± standard deviation. Sex (*F* = 6.117, *p* < 0.05), age (*F* = 10.392, *p* < 0.01), and self-care ability (*F* = 34.07, *p* < 0.01) had a significant impact on the level of understanding; educational level had no significant impact on level of understanding; there was no significant difference in the level of understanding between elderly people who lived in rural and urban areas. To further clarify the impact of different variables, we carried out multivariate regression analysis.

#### 3.3.2. Multivariate Linear Regression Analysis of Level of Understanding of COVID-19 among the Elderly Individuals

The linear regression analysis variable assignments are shown in Table 6. Table 7 shows that among the independent variables, sex, age, and self-care ability all passed the significance test at a level of *p* < 0.05 and that self-care ability had the most significant effect on the level of understanding of elderly individuals. The regression coefficient for sex was 0.371 (*t* = 2.365, *p* < 0.05), and the coefficient for self-care was 0.759 (*t* = 8.450, *p* < 0.001), indicating that sex and self-care had a significant positive effect on the level of risk cognition. The regression coefficient for age was −0.212 (*t* = −1.965, *p* < 0.05), indicating that age had a significant negative impact on the level of understanding. These results show that elderly individuals who were female, were of younger age and had better self-care ability had a higher level of risk cognition.

### 3.4. Influencing Factors of Protective Behaviors of Elderly Individuals Taken in Response to COVID-19

#### 3.4.1. Chi-Square Test for Protective Behaviors against COVID-19 Based on Different Population Characteristics

Regarding protective behavior against COVID-19, the participants’ sex $(\chi^{2}=18.265,p=0.000\;\left(<0.01\right)$), place of residence ($\chi^{2}=8.364,p=0.004\;(<0.01$)), level of understanding ($\chi^{2}=10.472,p=0.000\;\left(<0.01\right)$), information evaluation status ($\chi^{2}=24.333,p=0.000\;\left(<0.01\right)$), and age group ($\chi^{2}=7.746,p=0.021\;\left(<0.05\right)$) passed the significance test, showing statistical significance (Table 8).

#### 3.4.2. Logistic Regression Analysis of COVID-19 Protective Behaviors Based on Different Population Characteristics

The statistically significant indicators based on the chi-square test results in Table 8 were included in the multivariate regression model. Logistic multivariate regression was used to build the model, the −2 times log-likelihood ratio was 604.627, $\chi^{2}=$ 60.524, *p* < 0.001, and the overall test of the model was statistically significant. The logistic regression analysis variable assignments are shown in Table 9.

The results showed that sex (odds ratio (OR): 2.015, 95%; confidence interval (CI): 1.369–2.965; *p* < 0.001), place of residence (OR: 1.776; 95% CI: 1.198–2.634; *p* < 0.005), level of understanding (OR: 1.983; 95% CI: 1.325–2.967; *p* < 0.005) and information evaluation status (OR: 2.776; 95% CI: 1.824–4.224; *p* < 0.001) had significant effects on the participants’ protective behaviors. Being female, living in cities, and having positive information evaluations were associated with good behaviors. Age (OR: 1.097; 95% CI: 0.839–1.434; *p* > 0.01) was not significantly correlated with protective behaviors. (Table 10).

## 4. Discussion

In this paper, a questionnaire survey was applied to elderly individuals 60 years old and above to analyze the current level of understanding and coping behaviors with respect to COVID-19 among the elderly population and to study the influencing factors of both. The study found that (1) elderly individuals’ sex, age, and self-care ability were significantly correlated with their level of understanding, while place of residence and educational level were not significantly correlated. (2) Elderly individuals’ sex, place of residence, level of understanding and evaluation of COVID-19-related information were significantly correlated with their protective behaviors, while age was not significantly correlated. We discuss our findings in detail below.

### 4.1. Women Had a Higher Level of Understanding of COVID-19 than Did Men

It is observed that women had a higher level of understanding of COVID-19 than did men (Table 7). In general, men have better cognitive functioning than women [31]. However, we believe that with the continuous progress in gender equality policies, the educational level of Chinese women has continuously improved and that their economic status has gradually developed [32]. Driven by e-government and information and communications technology (ICT), women’s social equality at the information level has been dramatically enhanced [33]. Additionally, during the COVID-19 epidemic, most family members are living together because of closed community management. Due to their gender advantages regarding emotional expression [34], women have more persuasive communication skills [35], and they receive more information about COVID-19 within the family than men and have a higher level of understanding of COVID-19.

### 4.2. Age Is Negatively Correlated with the Level of Understanding of COVID-19

We found that age is negatively correlated with the level of understanding of COVID-19 (Table 7). People’s understanding level of risk is affected by their cognitive functioning. For the elderly, cognitive functioning gradually degrades with increasing age, which in turn affects memory and thinking, judgment and understanding, calculation and learning abilities, and language [36]. Elderly individuals of younger age have better cognitive functioning than elderly individuals of older age. Additionally, elderly individuals of younger age have a higher rate of social participation [37] and broader social contact [24]. Therefore, elderly individuals of younger age have more opportunities for exposure to COVID-19-related risk knowledge from the outside world, and their level of understanding is higher.

The age effect on the decline in self-care ability of elderly individuals of older age is common [38]. As age increases, elderly individuals’ level of self-care ability decreases. Elderly individuals with a gradually weakened level of self-care ability have fewer and fewer opportunities for social participation. Therefore, the opportunity to obtain information about COVID-19 will also decrease, which will reduce the level of understanding of COVID-19.

Human aging is an irreversible process. With the increase in age and decrease in self-care ability, people’s cognitive ability inevitably declines. During the COVID-19 epidemic, elderly individuals of older age and those with less self-care ability have lower levels of understanding with respect to the virus. Some elderly individuals who live at home alone and, thus, have insufficient opportunities for social participation have a poorer ability to obtain relevant knowledge of COVID-19 and are more susceptible to the virus.

### 4.3. Educational Level Is Not Significantly Associated with Understanding Level

As shown in Table 7, we observed that educational level is not significantly associated with understanding level. It is generally believed that elderly individuals with a high educational level have a higher level of understanding [23]. However, during the COVID-19 epidemic, the relationship between the educational level of elderly individuals and their level of understanding is not significant. There are three potential reasons for the inconsistency with existing research. First, the increase in information media, such as WeChat and Weibo, and the explosive spread of COVID-19 epidemic prevention and control information have led to increased public media exposure [39]. Under the psychological impact of the sudden public health event [40], elderly individuals have actively increased their awareness of COVID-19. Second, closed community management measures adopted by the government and family reunions during the Chinese Spring Festival enabled different family members to fully communicate regarding COVID-19, reducing the negative impact on the understanding of COVID-19 of elderly individuals with a low educational level. Due to the 2003 SARS epidemic, the Chinese government had learned from previous experience and adopted a fixed time, content, and format [39] for releasing COVID-19 epidemic prevention and control information to the public at a fixed time every day through various social media. Doing so has increased the opportunity for elderly individuals to understand COVID-19, compensating for the lack of understanding of COVID-19 among elderly individuals with low educational levels.

### 4.4. Place of Residence Is Not Significantly Associated with the Level of Understanding of COVID-19

Place of residence is not significantly associated with the level of understanding of COVID-19 as presented in Table 7. A related study in 2013 found that because of the different levels of convenience of health education [22], urban residents in China had higher levels of understanding of general influenza than rural residents. In this survey, elderly individuals living in urban areas did not have a significantly higher level of understanding of COVID-19 than those in rural areas, which is inconsistent with the conclusions of existing research. There are two possible reasons for this result. First, since 2013, China has implemented a 10-year “Beautiful Rural Construction” project. The central government and social funds have been invested in improving public service facilities and living environments in rural communities [41]. During this period, the vast majority of rural communities in China have built new public health service facilities, and the community health service network for rural residents has continued to improve. The content and level of medical services received by rural residents have gradually been approaching those received by urban residents [42]. In addition, through the newly established community health network, rural residents can obtain information about the epidemic and medical services relatively easily. These measures have gradually eliminated the gap between the rural and urban populations with regard to receiving public health services. Second, in terms of the source of COVID-19-related information for elderly individuals, their community and their children’s notifications are the main channels for obtaining COVID-19-related information. There are no distinct urban and rural differences in these channels.

### 4.5. Female Elderly Individuals Are More Likely to Take Effective Protective Actions

It is found that sex is significantly associated with protective behavior (Table 10). The existing literature reports that increased knowledge of infectious diseases is helpful for effective disease control [43] and that women take better protective measures than men [18]. During the COVID-19 epidemic, women’s level of understanding of COVID-19 risks is higher than that of men’s, and their protective behaviors are also better.

It can be speculated that as with political participation [44], women’s understanding of COVID-19 risks has affected their epidemic preventive measures, has affected their COVID-19 protection effects, and has ultimately affected their discourse power in epidemic prevention and control. An increase in the right to speak would bring an increase in status [45]. Therefore, it can also be speculated that with the improvement in women’s discourse power with respect to understanding of COVID-19 and protection, women’s status in the family is also improved.

### 4.6. Urban Elderly Individuals Are More Likely to Implement Effective Protective Behaviors

As shown in Table 10, we found that urban elderly individuals are more likely to implement effective protective behaviors. In general, a higher level of knowledge regarding infectious diseases is beneficial for effective disease control. Based on this survey, independent of living in urban or rural areas, the level of understanding of COVID-19 risks among elderly individuals was not significantly different. However, in the implementation of protective behaviors, the protective behaviors of elderly individuals living in cities are better. There are two possible reasons for this result. First, people with higher educational levels are more likely to accept health management advice and change poor behaviors [46]. The average educational level of the rural elderly population is lower than that of the urban elderly population, and the intention of rural elderly individuals to adopt COVID-19 protective behavior recommendations is also lower than that of their urban counterparts. The incidence of adverse health behaviors is relatively high [26]. Second, the scope of rural social interaction is relatively narrow. In mature stages of self-awareness, elderly individuals are more likely to form an inner self. They care more about their feelings, often adhere to their standards of behavior and beliefs and are less affected by the external environment [47]. Therefore, even with a better understanding of COVID-19, many elderly individuals still implemented protective behaviors while following their inner selves.

### 4.7. Information Disclourse Is Critical for Adopting Effective Protective Behaviors

It is suggested that elderly individuals with higher levels of understanding are more likely to have good behaviors with effective information disclosure (Table 10). Information disclosure has a positive role in promoting government trust [48]. Trust in the government can make the public more willing to disseminate and adopt information [49]. During the prevention and control phase of the COVID-19 epidemic, the government has been the controller and publisher of authoritative information in this public health crisis. Government information disclosure can effectively resolve the conflicting information between the government and the public and enhance people’s trust in governmental actions [50]. With trust in publicly available information from the government, elderly individuals are more willing to spread epidemic prevention and control information and take protective actions as directed.

Hence, it is suggested that during the prevention and control stage of this epidemic, the government must provide relevant information on COVID-19 and epidemic prevention and control and select the appropriate media to disclose such information in a timely manner based on the primary means by which the public, including the elderly population, receives information. When the control and prevention of the domestic epidemic have achieved preliminary effects, the government must fully disclose the prevention and control information regarding imported cases from abroad and remind the public to take related protective measures.

## 5. Conclusions

To understand the influencing factors of understanding of COVID-19 risks and coping behaviors with respect to COVID-19 among the elderly population, this study utilized snowball sampling to conduct a questionnaire survey on the elderly population using China as an example. The findings from this study demonstrate that (1) sex (*t* = 2.365, *p* < 0.05), age (*t* = −1.965, *p* < 0.05), and self-care ability (*t* = 8.450, *p* < 0.001) were significantly correlated with level of understanding of COVID-19. (2) Sex (OR: 2.015; 95% CI: 1.369–2.965; *p* < 0.001), level of understanding (OR: 1.983; 95% CI: 1.325–2.967; *p* < 0.005), place of residence (OR: 1.776; 95% CI: 1.198–2.634; *p* < 0.005) and evaluation of relevant information provided by the government (OR: 2.776; 95% CI: 1.824–4.224; *p* < 0.001) were significantly correlated with coping behaviors. The conclusions of this study can play a decisive role in relevant departments to formulate policies promptly and to implement COVID-19 prevention and control measures in a targeted manner to reduce the risk of infection in the elderly population.

The limitation of this study is that the article does not discuss the relationship between the attitudes of elderly individuals toward the epidemic and their response behaviors. It is hoped that follow-up studies increase the sample size to further explore the impact of elderly individuals’ attitudes toward public health events on their response behaviors. Due to the snowball sampling used in the selection of participants, the findings cannot be generalized and are limited to sub-population with homogeneous characteristics. In addition, since this is a cross-sectional study, the results are based on associations.

## Figures and Tables

**Table 1:** Sample population attributes.

Variable	Category	Number	Percentage
Sex	Male	221	43.5
	Female	287	56.5
Age groups	60–70 years old	239	47.0
	71–80 years old	185	36.4
	Over 80 years old	84	16.5
Educational level	Never attended school	104	20.5
	Elementary school	186	36.6
	Middle school	123	24.2
	High school	71	14.0
	Undergraduate/bachelor’s degree	18	3.5
	Postgraduate and above	6	1.2
Self-care ability	Completely independent	266	52.4
	Mostly independent	140	27.6
	Requires assistance but can provide some self-care	80	15.7
	Dependent on others	22	4.3
Place of residence	Urban	280	55.1
	Rural	228	44.9

**Table 2:** Descriptive statistics for the understanding level of the elderly participants.

Topic	Question	*n*	%
Epidemiologic features	Who do you think is vulnerable to COVID-19?	176	34.65
	What do you think are the main symptoms of people with COVID-19 infection?	79	15.6
	What are the currently identified routes of COVID-19 transmission?	208	40.9
Etiological characteristics	Which of the following options can be a source of COVID-19 infection?	143	28.1
Prevention and control measures	Which of the following measures do you think can prevent COVID-19 infection?	158	31.1
	Which of the following masks do you think are effective for preventing the spread of COVID-19?	134	26.4
	How many days do you think people who have been in close contact with COVID-19 patients need to be isolated?	305	60.04

**Table 3:** Response measures taken by the elderly participants (%).

Measure Taken	Never	Seldom	Occasionally	Frequently	Always
Effective preventive measures					
Wear a mask when going out	4.72	8.46	9.25	25.98	51.57
Disinfect the home	6.3	9.25	22.24	35.83	26.38
Open windows frequently to maintain indoor air circulation	3.35	5.51	12.4	37.6	41.14
Measure body temperature	8.07	18.7	19.49	27.95	25.79
Avoid visiting crowded areas and places with poor air circulation	8.27	7.87	8.66	28.54	46.65
Avoid visiting friends and family	6.69	8.27	11.02	25.79	48.23
Eat a balanced diet, quit drinking alcohol, and maintain adequate sleep and rest times	4.92	6.1	12.2	31.89	44.88
Actively obtain information and guidance on new developments, preventive measures, and anxiety relief	3.94	9.84	13.58	34.06	38.58
Unproven preventive measures					
Take traditional Chinese medicine	28.35	22.05	16.93	18.31	14.37
Take vitamins or supplements (such as royal jelly, ginseng, etc.)	24.02	20.28	19.88	22.05	13.78
Use antiviral drugs	31.1	20.87	16.73	19.88	11.42
Negative measures					
Avoid obtaining and discussing information related to the disease	31.3	16.54	14.17	22.24	15.75
Pretend the outbreak is not happening, that is, take no action or never think about it; no change in everyday life	40.16	15.94	14.96	15.55	13.39

**Table 4:** Evaluation of COVID-19-related information.

Factor	Content	Mean
Evaluation of relevant information	Disclosure of the disease was timely	3.87
	Disclosure of the disease was adequate	3.95
	Disclosure of the disease was authentic	3.95
	Overall satisfaction	3.92

**Table 5:** One-way analysis of variance (ANOVA) ($\bar{x}\;\pm \;s$).

Variable	Category	*n*	$\bar{x}\pm s$	*F*-Value	*p*-value
Sex	Male	221	2.14 ± 1.87	6.117	0.017 **
	Female	287	2.55 ± 1.85		
Age group	60–70	239	2.76 ± 1.78	10.392	0.000 ***
	71–80	185	2.03 ± 1.87		
	80	84	2.00 ± 1.91		
Educational level	Never attended school	104	1.93 ± 1.69	1.576	0.165
	Elementary school	186	2.56 ± 1.93		
	Middle school	123	2.42 ± 1.78		
	High school	71	2.42 ± 2.05		
	Undergraduate/bachelor’s degree	18	2.39 ± 1.88		
	Postgraduate and above	6	2.17 ± 1.84		
Self-care ability	Completely independent	266	3.05 ± 1.73	34.07	0.000 ***
	Mostly independent	140	1.95 ± 1.86		
	Requires assistance but can provide some self-care	80	1.08 ± 1.44		
	Dependent on others	22	1.50 ± 1.34		
Place of residence	Rural	280	2.28 ± 1.88	1.553	0.213
	Urban	228	2.48 ± 1.85		

Note: *** *p* < 0.01, ** *p* < 0.05; $\bar{x}\;\pm \;s$ refers to mean ± standard deviation.

**Table 6:** Linear regression analysis variable assignment for the understanding of coronavirus disease 2019 (COVID-19) among the elderly individuals.

Variables	Assignment
Understanding level	
Sex	1 = male, 2 = female
Age groups	1 = 60–70 years old 2 = 71–80 years old 3 = 80 years old or above
Educational level	1 = Never attended school 2 = Elementary school 3 = Junior high school 4 = High school 5 = Undergraduate/bachelor’s degree 6 = Postgraduate and above
Self-care ability	1 = Dependent on others 2 = Requires assistance but can provide some self-care 3 = Mostly independent 4 = Completely independent
Place of residence	1 = Rural, 2 = Urban

**Table 7:** Linear regression results of the understanding of COVID-19 among the elderly individuals.

Independent Variables	*B*	Standard Error	Beta	*t*	95% CI	
					Lower Limit	Upper Limit
Constant term	−0.660	0.523		−1.262	−1.688	0.367
Sex	0.367 **	0.155	0.098 **	2.365	0.062	0.673
Age group	−0.212 **	0.108	−0.084 **	−1.965	−0.423	0.000
Educational level	0.024	0.069	0.015	0.347	−0.112	0.160
Degree of self-care	0.759 ***	0.090	0.359 ***	8.450	0.583	0.936
Place of residence	0.181	0.155	0.048	1.168	−0.124	0.486

*** *p* < 0.01, ** *p* < 0.05.

**Table 8:** Chi-square test for protective behaviors against COVID-19 based on different population characteristics.

Variable	Category	Good Behaviors	Poor Behaviors	$\chi^{2}\;\mathrm{Value}$	*p*-Value
Sex	Male	118	103	18.265	0.000 ***
	Female	206	81		
Age group	60–70	160	79	7.746	0.021 **
	71–80	104	81		
	>80	60	24		
Place of residence	Rural area	163	117	8.364	0.004 ***
	Urban	161	67		
Level of risk cognition	Higher level of understanding	115	40	10.472	0.001 ***
	Low level of understanding	209	144		
Information evaluation status	Positive evaluation	109	258	24.333	0.000 ***
	Negative evaluation	66	75		

Note: *** *p* < 0.01, ** *p* < 0.05.

**Table 9:** Variable assignment table for COVID-19 protection behaviors based on different population characteristics.

Variable	Assignment
Behavior	0 = Poor behaviors 1 = Good behaviors
Sex	1 = Male 2 = Female
Age group	1 = 60–70 years old 2 = 71–80 years old 3 = 80 years old or above
Place of residence	1 = Rural 2 = Urban
Level of understanding	0 = Low level of understanding 1 = High level of understanding
Information evaluation status	0 = Negative evaluation 1 = Positive evaluation

**Table 10:** Logistic multivariate regression analysis of COVID-19 protective behaviors based on different population characteristics.

Independent Variables	*B*	Standard Error	*p*-Value	OR	95%CI	
					Lower Limit	Upper Limit
Sex	0.701 ***	0.197	0.000	2.015	1.369	2.965
Age group	0.093	0.137	0.498	1.097	0.839	1.434
Place of residence	0.575 **	0.201	0.004	1.776	1.198	2.634
Level of understanding	0.685 **	0.206	0.001	1.983	1.325	2.967
Information evaluation status	1.021 ***	0.214	0.000	2.776	1.824	4.224
Constant	−2.492 ***	0.522	0.000	0.083		

*** *p* < 0.01, ** *p* < 0.05.
